# Clinical and MRI efficacy of sc IFN β-1a tiw in patients with relapsing MS appearing to transition to secondary progressive MS: post hoc analyses of PRISMS and SPECTRIMS

**DOI:** 10.1007/s00415-019-09532-5

**Published:** 2019-09-26

**Authors:** Mark S. Freedman, Staley Brod, Barry A. Singer, Bruce A. Cohen, Brooke Hayward, Fernando Dangond, Patricia K. Coyle

**Affiliations:** 1 University of Ottawa and the Ottawa Hospital Research Institute, 501 Smyth Road, Ottawa, ON K1H 8L6 Canada; 2 Medical College of Wisconsin, Milwaukee, WI USA; 3 Missouri Baptist Medical Center, St. Louis, MO USA; 4 Northwestern University Feinberg School of Medicine, Chicago, IL USA; 5 EMD Serono, Inc., Rockland, MA USA; 6 EMD Serono, Inc., Billerica, MA USA; 7 Stony Brook University Medical Center, Stony Brook, NY USA

**Keywords:** Multiple sclerosis, Interferon β-1a, Relapsing–remitting MS, Secondary progressive MS

## Abstract

This study evaluated efficacy of subcutaneous (sc) interferon beta-1a (IFN β-1a) 44 µg 3 × weekly (tiw) in patients appearing to transition from relapsing–remitting multiple sclerosis (RRMS) to secondary progressive MS (SPMS). The PRISMS study included 560 patients with RRMS (EDSS 0–5.0; ≥ 2 relapses in previous 2 years), and the SPECTRIMS study included 618 patients with SPMS (EDSS 3.0–6.5 and ≥ 1-point increase in previous 2 years [≥ 0.5 point if 6.0–6.5]) randomly assigned to sc IFN β-1a 44 or 22 µg or placebo for 2–3 years, respectively. These post hoc analyses examined five subgroups of MS patients with EDSS 4.0–6.0: PRISMS (*n* = 59), PRISMS/SPECTRIMS (*n* = 335), PRISMS/SPECTRIMS with baseline disease activity (*n* = 195; patients with either ≥ 1 relapse within 2 years before baseline or ≥ 1 gadolinium-enhancing lesion at baseline), PRISMS/SPECTRIMS without baseline disease activity (*n* = 140), and PRISMS/SPECTRIMS with disease activity during the study (*n* = 202). In the PRISMS and PRISMS/SPECTRIMS subgroups, sc IFN β-1a delayed disability progression, although no significant effect was observed in PRISMS/SPECTRIMS subgroups with activity at baseline or activity during the study (regardless of baseline activity). In the PRISMS/SPECTRIMS subgroup, over year 1 (0–1) and 2 (0–2), sc IFN β-1a 44 µg tiw significantly reduced annualized relapse rate (*p* ≤ 0.001), and relapse risk (*p* < 0.05) versus placebo. Similar results were seen for the PRISMS/SPECTRIMS with baseline disease activity subgroup. Subcutaneous IFN β-1a reduced T2 burden of disease and active T2 lesions in the PRISMS/SPECTRIMS subgroups overall, with and without baseline activity. In conclusion, these post hoc analyses indicate that effects of sc IFN β-1a 44 µg tiw on clinical/MRI endpoints are preserved in a patient subgroup appearing to transition between RRMS and SPMS.

**Electronic supplementary material:**

The online version of this article (10.1007/s00415-019-09532-5) contains supplementary material, which is available to authorized users.

## Introduction

Relapsing–remitting multiple sclerosis (RRMS) is characterized by defined attacks separated by periods of stability. Over time, attacks become less frequent, while disability accumulates. Although the majority of patients with MS present with the relapsing form of the disease, relapses can continue to occur during the gradual transition to the progressive form of the disease, secondary progressive MS (SPMS) [1]. Disease severity is assessed using the Expanded Disability Status Scale (EDSS) score, which ranges from 0 (normal) to 10 (death due to MS) and is based on assessment of clinical deficits in various central nervous system functions. Patients with MS who have EDSS scores 4.0–6.0, while not limited to wheelchair or bed, have moderate disability indicative of disability progression [2]. Although there is no one agreed-upon definition of SPMS, it is usually defined as an initial relapsing–remitting disease course followed by progression with or without occasional relapses, minor remissions, and plateaus [3]. Patients with SPMS usually have an EDSS score 5.0–9.5 with impaired ambulation. Patients with an EDSS score 4.0–6.0 may be transitioning to SPMS; however, the disease course varies between patients [2, 4].

PRISMS (Prevention of Relapses and disability by Interferon beta-1a Subcutaneously in Multiple Sclerosis), a double-blind, placebo-controlled study, demonstrated that subcutaneous (sc) interferon beta-1a (IFN β-1a) three times weekly (tiw) significantly reduced relapses and active T2 lesions over 2 years in patients with active (with relapses and/or evidence of new magnetic resonance imaging [MRI] activity [5]) RRMS [6]. Disability progression was significantly delayed by sc IFN β-1a 44 µg tiw in the overall population, and in the prespecified subgroup with baseline EDSS score > 3.5 [6]. SPECTRIMS (Secondary Progressive Efficacy Clinical Trial of Recombinant Interferon beta-1a in MS), a double-blind, placebo-controlled study, demonstrated that sc IFN β-1a tiw reduced relapses and active T2 lesions over 3 years among patients with SPMS [7]. Disability progression was not significantly delayed in the overall population, although a greater, non-significant effect was seen in post hoc analyses of patients who had experienced a relapse ≤ 2 years before the study [7]. Both PRISMS and SPECTRIMS included patients with advanced disease at baseline, as well as patients experiencing ongoing disease activity.

While the past two decades have seen numerous effective therapies developed to reduce disease activity in RRMS, most therapies have not been evaluated specifically in patients with confirmed SPMS or in patients who are in the loosely defined transition period between RRMS and SPMS, and effective treatment and clinical management are still lacking [4, 8-13]. Given the positive results seen with sc IFN β-1a tiw in the high-EDSS population of PRISMS and the subgroup of patients in SPECTRIMS with recent relapses, patients from these two studies with similar disease characteristics were pooled to evaluate the effects of sc IFN β-1a tiw in this unique cohort.

## Methods

### Study design

In the PRISMS trial, patients with RRMS were randomly assigned to sc IFN β-1a tiw or placebo for 2 years [6]. A total of 560 patients between 18 and 50 years of age, with a history of > 2 relapses in the previous 2 years and an EDSS score of 0–5.0, were randomized and received treatment. Diagnosis of RRMS was based on the Poser criteria [14]. The primary endpoint was the number of relapses over 2 years. All patients had proton density (PD)/T2-weighted scans at baseline and twice yearly [15]. MRI endpoints in the overall PRISMS population included burden of disease (total area of MS lesions identified on a PD/T2 scan) and active (new, recurrent, and enlarging) T2 lesions. Other efficacy measures included disability progression, defined as an increase in EDSS score of ≥ 1 point sustained over at least 3 months [16].

In SPECTRIMS, 618 patients with SPMS (EDSS score increase of ≥ 1 point within the last 2 years [≥ 0.5 points if baseline EDSS score was 6.0–6.5]) and baseline EDSS score 3.0–6.5 were randomly assigned to receive sc IFN β-1a tiw or placebo for 3 years [7]. Cranial MRI scans were performed at baseline and twice yearly [17]. The primary endpoint was time to confirmed disability progression, defined as an increase from baseline in EDSS score of at least 1 point (or 0.5 points if baseline EDSS score was ≥ 6.0), confirmed 3 months later with no intervening score lower than the minimum required level. Additional clinical endpoints included relapse count and time to first relapse. MRI endpoints in the entire SPECTRIMS population included burden of disease and number of active T2 lesions [17].

### Exploratory analysis of PRISMS high-EDSS subgroup

A predefined subgroup of PRISMS included patients with active but advanced disease, characterized by EDSS 4.0–5.0 at baseline and > 2 relapses in the previous 2 years (defined as PRISMS subgroup). Exploratory analysis of this subgroup over 2 years included assessment of the number of relapses, patients free of relapse, time to first relapse, time to 3-month confirmed disability progression (increase of ≥ 1 point in EDSS score), T2 burden of disease, and active T2 lesions.

### Post hoc analyses of pooled subgroups from PRISMS and SPECTRIMS

Post hoc analyses examined the treatment effect of sc IFN β-1a 44 μg tiw versus placebo in a pooled subgroup of patients from PRISMS and SPECTRIMS with baseline EDSS scores 4.0–6.0 (defined as PRISMS/SPECTRIMS subgroup). To identify a subset of patients with advanced but active disease, the PRISMS/SPECTRIMS subgroup was then refined to include patients within this disability range who had either ≥ 1 relapse within 2 years before baseline or ≥ 1 gadolinium-enhancing (Gd) lesion at baseline, referred to as the PRISMS/SPECTRIMS with baseline disease activity subgroup; patients without active disease are referred to as the PRISMS/SPECTRIMS without baseline disease activity subgroup. Post hoc analyses were also conducted for 3-month confirmed EDSS progression on a small subset of patients who had disease activity (≥1 relapse) during the study (defined as PRISMS/SPECTRIMS with disease activity during the study subgroup, regardless of baseline activity) to examine the pattern of progression that may be due to relapse activity. Both trials included sc IFN β-1a 44 and 22 μg tiw treatment arms; as 44 μg is most commonly used, the analyses presented here compare only this dose to placebo. The following endpoints were investigated in all three subgroups: annualized relapse rate (ARR) over year 1 (0–1) and year 2 (0–2), time to relapse over 2 years, risk of relapse at 1 and 2 years, 3- and 6-month confirmed disability progression (EDSS score increase of ≥ 1 point [≥ 0.5 points if baseline EDSS score was ≥ 6.0]) at 1 and 2 years, mean number of active T2 lesions over 2 years (new, recurring, and newly enlarging T2 lesions), and burden of disease (total T2 lesion area) at 1 and 2 years.

### Statistical analyses

For the exploratory analysis of the PRISMS subgroup, comparisons were made between the subgroup who received sc IFN β-1a 44 μg tiw and those who received placebo. Independent sample *t* test was used to compare the number of relapses over time. Cochran–Mantel–Haenszel chi-square test was used to compare the percentage of patients who were relapse free. Between-treatment differences for time to first relapse and time to confirmed disability by 1 point on EDSS were compared using log-rank tests. Analysis of variance on the ranks with effects for baseline EDSS subgroup, center, and interaction between treatment and baseline EDSS subgroup was used to compare treatment groups for T2 burden of disease and the number of active T2 lesions.

For the post hoc analyses of pooled PRISMS/SPECTRIMS patients, sc IFN β-1a 44 μg tiw was compared with placebo in each subgroup (overall, patients with baseline disease activity, and patients without baseline disease activity). Hazard ratios (HRs), confidence intervals (CIs), and *p* values based on Cox proportional hazards model were used to compare between-treatment differences for risk of relapse, time to first relapse, and time to 3-month confirmed EDSS progression over 1 and 2 years. For ARR comparisons, *p* values were based on negative binomial regression. All comparisons were adjusted for the number of relapses within 2 years prior, age group (< 40 vs ≥ 40 years), and baseline burden of disease (with adjustment for baseline EDSS). T2 burden of disease *p* values at 6, 12, and 24 months were based on ranked analysis of covariance by adjusting for number of relapses within prior 2 years, age group (< 40 vs ≥ 40 years), baseline EDSS, baseline burden of disease, and derived using the Wilcoxon rank-sum test. The *t* test was used to compare treatment difference in the number of active T2 lesions. Number of T2 lesions was not measured at baseline in the SPECTRIMS study and thus not analyzed in all three subgroups.

## Results

### PRISMS subgroup

In the PRISMS trial (*n* = 371), 59 patients had a high EDSS score (4.0–5.0; Tables 1 and 2). As in the overall trial population [6], PRISMS patients with EDSS 4.0–5.0 treated with sc IFN β-1a (*n* = 31) had significantly reduced relapses, T2 burden of disease, number of active T2 lesions, and delayed time to confirmed 3-month disability progression versus placebo (*n* = 28) (Table 3).

**Table 1:** Patients with high EDSS (4.0‒6.0) in the SPECTRIMS and PRISMS studies

Treatment received	PRISMS (*n* = 371)		SPECTRIMS (*n* = 409)		PRISMS/SPECTRIMS pooled (*n* = 335)		
	*N* per treatment	*n* (% with high EDSS)	*N* per treatment	*n* (% with high EDSS)	*N* per treatment	*n* (% from PRISMS)	*n* (% from SPECTRIMS)
Placebo	187	28 (15.0)	205	136 (66.3)	164	28 (17.1)	136 (82.9)
sc IFN β-1a 44 µg tiw	184	31 (16.8)	204	140 (68.6)	171	31 (18.1)	140 (81.9)

*EDSS* Expanded Disability Status Scale, *IFN β-1a* interferon beta-1a, *sc* subcutaneous, *tiw* three times weekly

**Table 2:** Baseline characteristics in the high-EDSS subgroups

	PRISMS (*N* = 59)		PRISMS/SPECTRIMS			
			All patients (*n* = 335)		With baseline disease activity^a^ (*n* = 195)	
Characteristic	Placebo (*n* = 28)	sc IFN β-1a 44 μg tiw (*n* = 31)	Placebo (*n* = 164)	sc IFN β-1a 44 μg tiw (*n* = 171)	Placebo (*n* = 92)	sc IFN β-1a 44 μg tiw (*n* = 103)
Age, years						
Mean (SD)	37.6 (8.0)	36.6 (7.6)	41.5 (7.3)	41.2 (7.3)	40.0 (7.4)	39.4 (7.2)
Female sex, *n* (%)	24 (86)	17 (55)	108 (65.9)	107 (62.6)	62 (67.4)	66 (64.1)
Time since diagnosis, years^b^						
Mean (SD)	8.9 (6.4)	9.2 (6.4)	13.3 (7.3)	12.4 (7.0)	12.0 (7.5)	10.8 (6.4)
EDSS score at baseline^b^						
Mean (SD)	4.4 (0.5)	4.5 (0.6)	5.2 (0.8)	5.2 (0.8)	5.1 (0.8)	5.0 (0.8)
Relapses in previous 2 years^b^						
Mean (SD)	3.2 (1.4)	2.8 (1.1)	1.3 (1.5)^c^	1.3 (1.5)^d^	2.2 (1.4)	2.1 (1.4)
Burden of disease,^b^ mm^2^						
Mean (SD)	4124.7 (3973.1)	4110.2 (3324.8)	4459.6 (3775.5)	4441.6 (4213.7)	4601.8 (4082.3)	4879.7 (4608.9)

*EDSS* Expanded Disability Status Scale, *IFN β-1a* interferon beta-1a, *sc* subcutaneous, *SD* standard deviation, *tiw* three times weekly

^a^Active disease defined as having ≥ 1 relapse within 2 years before baseline or ≥ 1 gadolinium-enhancing lesion at baseline

^b^Equals the number of patients with available data

^c^*n* = 163

^d^*n* = 170

**Table 3:** Clinical and MRI endpoints: PRISMS subgroup

	Placebo (*n* = 28)	sc IFN β-1a 44 µg tiw (*n* = 31)	*p*
Number of relapses at year 2			
Mean (SD)	3.1 (1.84)	1.2 (1.20)	
Median	3.0	1.0	< 0.0001^a^
Patients relapse free at year 2, *n* (%)	2 (7.1)	10 (32.3)	0.0177^b^
Time to first relapse			
Median, days (months)	84 (2.8)	324 (10.6)	0.0012^c^
Time to 3-month confirmed disability progression			
First quartile, days (months)	218 (7.2)	638 (21.0)	0.0481^c^
T2 burden of disease, % change			
Median (mean)	5.4 (12.2)	–6.9 (0.7)	0.0207^d^
Active T2 lesions per patient per scan			
Median (mean)	1.9 (2.6)	0.5 (0.9)	0.0002^d^

*EDSS* Expanded Disability Status Scale, *IFN β-1a* interferon beta-1a, *MRI* magnetic resonance imaging, *sc* subcutaneous, *SD* standard deviation, *tiw* three times weekly

^a^Independent sample *t* test

^b^Cochran–Mantel–Haenszel chi-square test

^c^Log-rank test

^d^Analysis of variance on the ranks with effects for baseline EDSS subgroup, center, and interaction between treatment and baseline EDSS subgroups

### PRISMS/SPECTRIMS subgroup

A total of 335 patients with EDSS 4.0–6.0 were included in the pooled PRISMS/SPECTRIMS subgroup (PRISMS, *n* = 59; SPECTRIMS, *n* = 276; Table 1). Patients in the PRISMS/SPECTRIMS subgroup were slightly older than those in the PRISMS subgroup, with longer duration of disease, higher burden of disease, and fewer relapses in the previous 2 years (Table 2). Within the PRISMS/SPECTRIMS subgroup, outcomes for patients with active disease (≥ 1 relapse in prior 2 years or ≥ 1 Gd lesion at baseline; *n* = 195 [58%] patients) versus those with no disease activity at baseline (no Gd lesions and no relapse in prior 2 years) were also examined.

#### Relapses

**Fig. 1:**
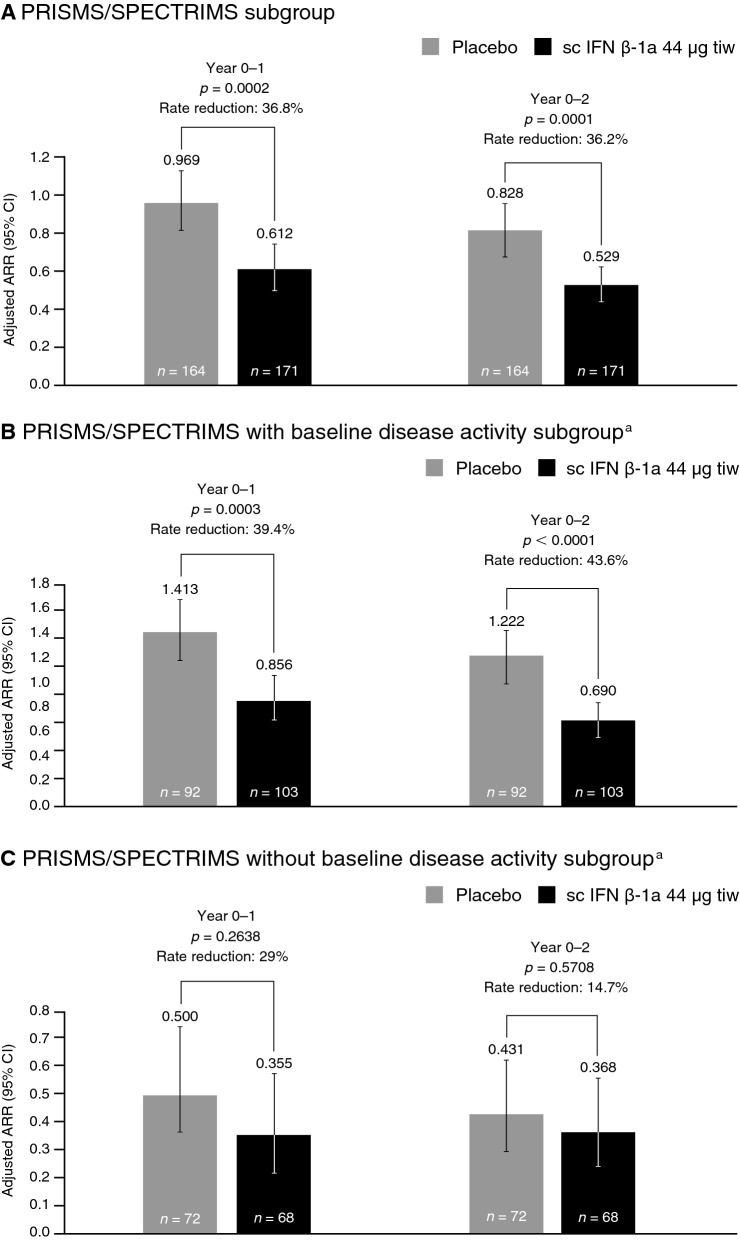
ARR over 1 and 2 years (PRISMS/SPECTRIMS). ^a^Active disease defined as having either ≥ 1 relapse within 2 years before baseline or ≥ 1 gadolinium-enhancing lesion at baseline. *p* values based on negative binomial regression, adjusted for number of relapses within 2 years prior, age group (< 40 vs ≥ 40 years), and baseline burden of disease (in the EDSS 4.0–6.0 subgroup, adjustment was also made for baseline EDSS). *ARR* annualized relapse rate, *CI* confidence interval, *EDSS* Expanded Disability Status Scale, *IFN β-1a* interferon beta-1a, *sc* subcutaneous, *tiw* three times weekly

**Fig. 2:**
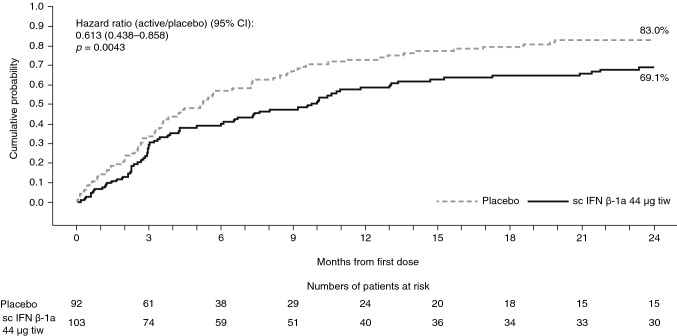
Time to first relapse over 2 years in the PRISMS/SPECTRIMS with baseline disease activity^a^ subgroup. ^a^Active disease defined as having either ≥ 1 relapse within 2 years before baseline or ≥ 1 gadolinium-enhancing lesion at baseline. Hazard ratio and *p* value based on Cox proportional hazards model, adjusted for number of relapses within 2 years prior, age group (<40 vs ≥ 40 years), and baseline burden of disease. *CI* confidence interval, *EDSS* Expanded Disability Status Scale, *IFN β-1a* interferon beta-1a, *sc* subcutaneous, *tiw* three times weekly

In PRISMS/SPECTRIMS patients with high EDSS (4.0–6.0), sc IFN β-1a significantly reduced ARR versus placebo at year 1 and year 2 (Fig. 1a). The reduction in ARR was significant in the subgroup with active disease at baseline (Fig. 1b), but not significant in the subgroup without baseline disease activity (Fig. 1c). Treatment with sc IFN β-1a significantly lowered the risk of relapse versus placebo over year 1 and year 2 in the PRISMS/SPECTRIMS subgroup and the PRISMS/SPECTRIMS with baseline disease activity subgroup (Table 4). Subcutaneous IFN β-1a significantly delayed the time to first relapse over 2 years’ treatment (*p* = 0.0043) in the PRISMS/SPECTRIMS with baseline disease activity subgroup (Fig. 2).

**Table 4:** Risk of relapse versus placebo over 1 and 2 years (PRISMS/SPECTRIMS)

Risk of relapse	PRISMS/SPECTRIMS		PRISMS/SPECTRIMS with baseline disease activity^a^		PRISMS/SPECTRIMS without baseline disease activity	
	Placebo (*n* = 164)	sc IFN β-1a 44 µg tiw (*n* = 171)	Placebo (*n* = 92)	sc IFN β-1a 44 µg tiw (*n* = 103)	Placebo (*n* = 72)	sc IFN β-1a 44 µg tiw (*n* = 68)
Year 1						
Risk of relapse vs placebo^b^						
Patients with relapse, *n* (%)	90 (54.9)	77 (45.0)	66 (71.7)	59 (57.3)	24 (33.3)	18 (26.5)
Patients without relapse, *n* (%)	74 (45.1)	94 (55.0)	26 (28.3)	44 (42.7)	48 (66.7)	50 (73.5)
HR vs placebo (95% CI)		0.696 (0.511–0.947)		0.659 (0.461–0.942)		0.759 (0.411–1.402)
* p* value		0.0213		0.0223		0.3789
Year 2						
Risk of relapse vs placebo^b^						
Patients with relapse, *n* (%)	106 (64.6)	96 (56.1)	76 (82.6)	69 (67.0)	30 (41.7)	27 (39.7)
Patients without relapse, *n* (%)	58 (35.4)	75 (43.9)	16 (17.4)	34 (33.0)	42 (58.3)	41 (60.3)
HR vs placebo (95% CI)		0.696 (0.525–0.923)		0.613 (0.438–0.858)		0.866 (0.511–1.466)
* p* value		0.0119		0.0043		0.5917

*CI* confidence interval, *EDSS* Expanded Disability Status Scale, *HR* hazard ratio, *IFN β-1a* interferon beta-1a, *sc* subcutaneous, *tiw* three times weekly

^a^Active disease defined as having either ≥ 1 relapse within 2 years before baseline or ≥ 1 gadolinium-enhancing lesion at baseline

^b^HR and *p* value based on Cox proportional hazards model, adjusted for number of relapses within 2 years prior, age group (<40 vs ≥ 40 years), and baseline burden of disease (in the EDSS 4.0–6.0 subgroup, adjustment was also made for baseline EDSS)

#### Disability progression

**Fig. 3:**
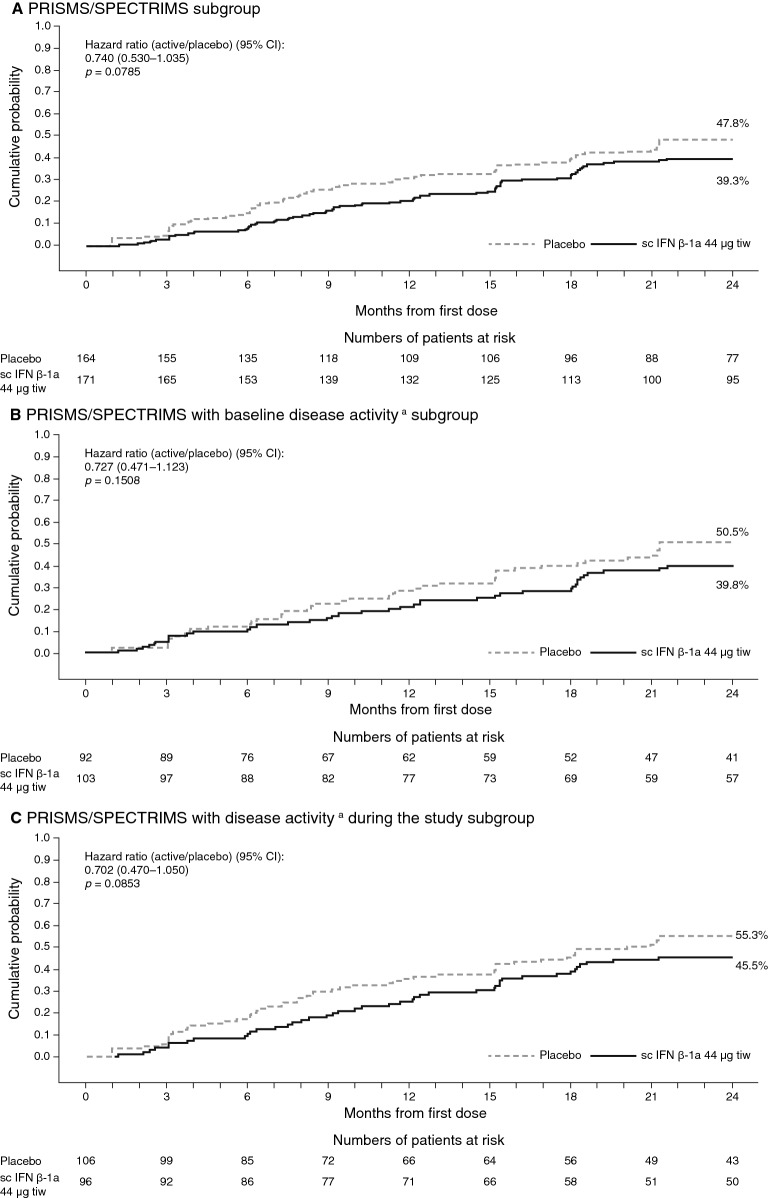
Time to 3-month confirmed EDSS progression over 2 years (PRISMS/SPECTRIMS). ^a^Active disease at baseline defined as having either ≥ 1 relapse within 2 years before baseline or ≥ 1 gadolinium-enhancing lesion at baseline, and active disease during the study defined as ≥ 1 relapse during the study (regardless of baseline activity). Hazard ratio (vs placebo) and *p* value estimated from a Cox proportional hazards model, adjusted for number of relapses within 2 years prior, age group (< 40 vs ≥ 40 years), and baseline burden of disease. *CI* confidence interval, *EDSS* Expanded Disability Status Scale, *IFN β-1a* interferon beta-1a, *sc* subcutaneous, *tiw* three times weekly

In the PRISMS/SPECTRIMS subgroup, sc IFN β-1a was associated with a lower risk of 3-month EDSS progression versus placebo over 1 year [HR 0.654 (95% CI 0.429–0.997); *p* = 0.0486] and over 2 years, although this did not achieve statistical significance regardless of baseline disease activity or activity during the study (Fig. 3). Numerically fewer patients treated with sc IFN β-1a versus placebo had 3-month EDSS progression (year 1, 23% vs 29%; year 2, 38% vs 48%). Over 2 years, the time to first EDSS progression was delayed with sc IFN β-1a treatment; however, the HR was similar between all three subgroups (Fig. 3). There were no differences in the time to 6-month confirmed disability progression for patients treated with sc IFN β-1a compared with placebo over 2 years in the PRISMS/SPECTRIMS with baseline disease activity subgroup [HR 0.995 (95% CI 0.597–1.657); *p* = 0.9832] or the PRISMS/SPECTRIMS with disease activity during the study subgroup [HR 0.762 (95% CI 0.490–1.187); *p* = 0.2293].

#### MRI endpoints

**Fig. 4:**
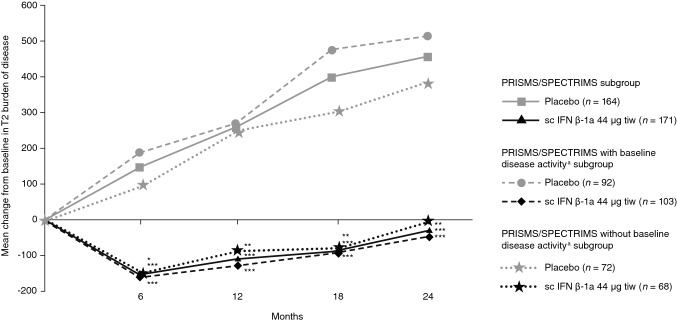
T2 burden of disease at 6, 12, and 24 months in the PRISMS/SPECTRIMS subgroup, PRISMS/SPECTRIMS with baseline disease activity^a^ subgroup, and PRISMS/SPECTRIMS without baseline disease activity subgroup. *p* values are for between-treatment differences in change from baseline. ^a^Active disease defined as having either ≥ 1 relapse within 2 years before baseline or ≥ 1 gadolinium-enhancing lesion at baseline. *p < 0.05 based on ranked analysis of covariance by adjusting for number of relapse within prior 2 years, age group (<40 vs ≥ 40 years), baseline EDSS, baseline burden of disease, and by Wilcoxon rank-sum test. **p < 0.005 based on ranked analysis of covariance by adjusting for number of relapse within prior 2 years, age group (< 40 vs ≥ 40 years), baseline EDSS, baseline burden of disease, and by Wilcoxon rank-sum test. ***p ≤ 0.0001 based on ranked analysis of covariance by adjusting for number of relapse within prior 2 years, age group (<40 vs ≥ 40 years), baseline EDSS, baseline burden of disease, and by Wilcoxon rank-sum test. *EDSS* Expanded Disability Status Scale, *IFN β-1a* interferon beta 1-a, *sc* subcutaneous, *tiw* three times weekly

Subcutaneous IFN β-1a significantly reduced the T2 burden of disease from baseline compared with placebo through year 2 in all PRISMS/SPECTRIMS subgroups with EDSS 4.0–6.0 (Fig. 4) Compared with placebo, sc IFN β-1a also significantly reduced the mean numbers of active T2 lesions at 6, 12, and 24 months in the overall PRISMS/SPECTRIMS subgroup and PRISMS/SPECTRIMS with baseline disease activity subgroup, but not in the PRISMS/SPECTRIMS without baseline disease activity subgroup (Supplementary Fig. 1).

## Discussion

In this post hoc analysis of the pooled subgroup of patients with EDSS 4.0–6.0 from the PRISMS RRMS trial and the SPECTRIMS SPMS trial, sc IFN β-1a 44 µg tiw was effective at reducing relapses and T2 lesion activity versus placebo. Greater efficacy was seen in patients with active disease at baseline (≥ 1 relapse in prior 2 years or ≥ 1 Gd lesion). In patients with high EDSS from the PRISMS trial, sc IFN β-1a delayed disability progression; in the subgroup of patients from both trials, sc IFN β-1a significantly delayed disease progression over 1, but not 2 years. However, no significant effect on delaying further disease progression was seen in the PRISMS/SPECTRIMS with baseline disease activity subgroup. The HR for 3-month disability progression was similar between the PRISMS/SPECTRIMS subgroups. Taken together, these data suggest that baseline disease activity may help identify those patients who could have relapses or radiological progression without treatment.

For the PRISMS/SPECTRIMS without baseline disease activity subgroup, no statistically significant effects of sc IFN β-1a were observed on ARR; however, treatment reduced T2 lesion activity and number in this subgroup, although the low patient number in this subgroup may have influenced the result. Separation between treated and untreated groups in terms of time to disability progression could be seen early in the treatment course for this subgroup, with continued separation over 2 years, although statistical significance was not shown. These results are in line with the overall SPECTRIMS study in which inflammatory and radiological components of MS were more affected by sc IFN β-1a treatment than was disability progression [7].

The relationship between relapses and disability progression in RRMS has not been not fully elucidated. In patients with RRMS, relapses not only affect EDSS score in the short term [18, 19] but also have been shown to predict future confirmed disability progression [20]. However, other research in patients with more advanced disease has shown a lack of association between relapses and disability [21]. Some studies have suggested that once patients achieved a clinical threshold of disability (EDSS score of 4.0), disability progression was not significantly affected by relapses [22]. The results for the PRISMS and PRISMS/SPECTRIMS subgroups from this study are consistent with relapses having a greater effect on disability.

Some patients with MS may enter a period of fewer interactions with their healthcare provider or withdrawal of disease-modifying drugs (DMDs) as their disability accumulates and they transition to SPMS [23]. These changes in care and treatment are sometimes due to the perception of providers that there are no effective treatment options for patients who appear to be transitioning to SPMS. However, as shown in this study, patients with moderate disability can still experience clinical and MRI benefits from treatment.

Findings have been inconsistent regarding the ability of DMDs to delay disability progression in patients with RRMS with higher EDSS or in patients with SPMS regardless of relapse activity. Four large-scale studies assessed the effectiveness of IFN β in patients with SPMS [3, 24]. Among these IFN β studies, the European SPMS (EUSPMS) trial was the only trial to show a positive effect of treatment on the accumulation of irreversible disability progression [3, 24]. The differences in treatment benefit within these studies could be due to the different patient populations included. For example, placebo patients in the North American SPMS (NASPMS) trial progressed less than both placebo and active treatment groups in the EUSPMS trial, even though the inclusion criteria were comparable [25, 26]. Thus, patients participating in the EUSPMS trial were more likely closer to the relapsing phase of MS, while patients in the NASPMS trial were further along in the course of the disease [3]. Evidence is also inconclusive for the effects of other DMDs in patients with high EDSS or SPMS. Natalizumab treatment effect seemed to favor patients with RRMS who have lower baseline EDSS scores (≤ 3.5) over those with higher scores [10]; furthermore, natalizumab did not delay progression of ambulatory disability in patients with SPMS (in a cohort with baseline EDSS score 3.0–6.5 [mean 5.6], 29% of whom had relapses within the previous 2 years) [11]. In a subgroup analysis of the FREEDOMS study, fingolimod showed a 68% reduction in the odds of disability progression in those with higher baseline EDSS scores (> 3.5) versus a 23% reduction among those with lower scores; however, the relapse activity in the two subgroups was not described [12]. In a subgroup analysis of the TEMSO trial, teriflunomide 14 mg showed a trend towards a greater effect on the risk of disability progression in patients with higher baseline EDSS scores (> 3.5) compared with those with lower scores; ARR was reduced most in patients with lower EDSS at baseline [13].

It is important to note that the PRISMS/SPECTRIMS subgroup described here included patients with SPMS from the SPECTRIMS trial, which failed to meet the primary endpoint of delaying disability progression. Most of the advances over the past two decades have been limited to patients with RRMS, with few treatments showing efficacy in slowing the rate of disability progression, specifically in patients with SPMS, whose disease has accumulated further.

Examinations of treatment efficacy in patients with moderate disability are of interest in light of the developing treatment outlook for patients with progressive disease. Two drugs, the sphingosine-1-phosphate receptor modulator siponimod and a purine antimetabolite, Cladribine tablets, were recently approved by the FDA for the treatment of adults with relapsing forms of MS, including SPMS with active disease [27, 28]. In a phase III study, siponimod significantly reduced risk of 3-month confirmed disability progression by 21% in patients with SPMS and reduced the ARR (0.07 [95% CI 0.06–0.09]) compared with placebo (0.16 [95% CI 0.12–0.21]). Further subgroup analysis identified favorable effects of siponimod versus placebo on the HR of 3-month disease progression in patients who had superimposed relapses in the 2 years before the study (HR 0.67 [95% CI 0.49–0.91]), which suggests that patients with active SPMS received a greater benefit from treatment with siponimod compared with patients with lower activity (HR 0.87 [95% CI 0.68–1.11]) [9]. In the phase III CLARITY trial, Cladribine tablets 3.5 mg/kg reduced ARR by 57.6% versus placebo (*p* < 0.001) in patients with RRMS, and reduced risk of 3-month disability progression (HR 0.69 [95% CI 0.49‒0.96]) [29]. In post hoc analyses of the CLARITY trial in which baseline EDSS score ≥ 3.5 was used as a proxy for active SPMS, Cladribine tablets reduced ARR versus placebo (relative risk 0.43 [95% CI 0.30‒0.62; *p* < 0.001), and 49% of patients treated with Cladribine tablets achieved no evidence of disease activity compared with 17% of patients who received placebo (odds ratio 4.51 [95% CI 2.65‒7.69]; *p* < 0.0001), indicating efficacy in patients with more advanced disease [30, 31]. In addition, the approved indications for other DMDs have been recently updated to include clinically isolated syndrome and active SPMS, and additional updates are expected [32, 33]. These expanded indications may be due to the recognition by regulatory agencies that clinically isolated syndrome, RRMS, and SPMS with relapses are all part of a spectrum of active disease and treatment is warranted at each stage.

The present research is limited by its post hoc nature. The selected patient subgroups having the characteristics of interest made up a small part of the populations from each of the source trials. Furthermore, our analysis did not include stratification of efficacy by patient factors, such as age and sex. Age may be an important predictor of efficacy, as demonstrated in a recent meta-analysis of randomized, blinded clinical trials of MS DMDs against placebo or active comparator, in which the efficacy of immunomodulatory DMDs was found to decrease with age [34]. Although our analysis did not include analysis by sex, a treatment-by-sex interaction was observed in female patients in the SPECTRIMS trial, showing a delay in progression compared with placebo with both sc IFN β-1a doses (*p* = 0.006 for 44 µg and *p* = 0.038 for 22 µg), whereas no difference was observed in male patients [7]. An additional limitation is in the lack of a clear definition of “transition” from RRMS to SPMS, and the difficulty of making this assessment within the confines of clinical trials of relatively short duration.

Overall, a similar magnitude of effect was observed for the overall PRISMS/SPECTRIMS subgroup and PRISMS/SPECTRIMS with baseline disease activity subgroup. While efforts were made to select a population consisting of patients from both trials with similar baseline characteristics, it should be noted that the trials had different entry criteria and reported discordant results of disability progression. There are also caveats while extrapolating these results to the modern MS patient population, as higher relapse rates were seen in placebo in PRISMS and SPECTRIMS than have been reported in more recent trials.

These post hoc analyses suggest that treatment with sc IFN β-1a 44 µg tiw effectively reduced relapses, burden of disease, T2 lesions, and in some cases, delayed disability progression in a subgroup of MS patients appearing to transition from RRMS to SPMS. Such patients with active disease and continued disability worsening may still derive some benefit from continued treatment with sc IFN β-1a.

## Electronic supplementary material

Below is the link to the electronic supplementary material.

Supplementary file1 (PDF 801 kb)

## References

[1] Lassmann H, van Horssen J, Mahad D (2012). Progressive multiple sclerosis: pathology and pathogenesis. Nat Rev Neurol.

[2] Kurtzke JF (1983). Rating neurologic impairment in multiple sclerosis: an expanded disability status scale (EDSS). Neurology.

[3] Rovaris M, Confavreux C, Furlan R, Kappos L, Comi G, Filippi M (2006). Secondary progressive multiple sclerosis: current knowledge and future challenges. Lancet Neurol.

[4] Lorscheider J, Buzzard K, Jokubaitis V, Spelman T, Havrdova E, Horakova D, Trojano M, Izquierdo G, Girard M, Duquette P, Prat A, Lugaresi A, Grand'Maison F, Grammond P, Hupperts R, Alroughani R, Sola P, Boz C, Pucci E, Lechner-Scott J, Bergamaschi R, Oreja-Guevara C, Iuliano G, Van Pesch V, Granella F, Ramo-Tello C, Spitaleri D, Petersen T, Slee M, Verheul F, Ampapa R, Amato MP, McCombe P, Vucic S, Sanchez Menoyo JL, Cristiano E, Barnett MH, Hodgkinson S, Olascoaga J, Saladino ML, Gray O, Shaw C, Moore F, Butzkueven H, Kalincik T, MS Base Study Group (2016). Defining secondary progressive multiple sclerosis. Brain.

[5] Lublin FD, Reingold SC, Cohen JA, Cutter GR, Sorensen PS, Thompson AJ, Wolinsky JS, Balcer LJ, Banwell B, Barkhof F, Bebo B, Calabresi PA, Clanet M, Comi G, Fox RJ, Freedman MS, Goodman AD, Inglese M, Kappos L, Kieseier BC, Lincoln JA, Lubetzki C, Miller AE, Montalban X, O'Connor PW, Petkau J, Pozzilli C, Rudick RA, Sormani MP, Stuve O, Waubant E, Polman CH (2014). Defining the clinical course of multiple sclerosis: the 2013 revisions. Neurology.

[6] PRISMS Study Group (1998). Randomised double-blind placebo-controlled study of interferon beta-1a in relapsing/remitting multiple sclerosis. Lancet.

[7] SPECTRIMS Study Group (2001). Randomized controlled trial of interferon-beta-1a in secondary progressive MS: clinical results. Neurology.

[8] Ontaneda D, Fox RJ, Chataway J (2015). Clinical trials in progressive multiple sclerosis: lessons learned and future perspectives. Lancet Neurol.

[9] Kappos L, Bar-Or A, Cree BAC, Fox RJ, Giovannoni G, Gold R, Vermersch P, Arnold DL, Arnould S, Scherz T, Wolf C, Wallstrom E, Dahlke F, Investigators Expand Clinical (2018). Siponimod versus placebo in secondary progressive multiple sclerosis (EXPAND): a double-blind, randomised, phase 3 study. Lancet.

[10] Hutchinson M, Kappos L, Calabresi PA, Confavreux C, Giovannoni G, Galetta SL, Havrdova E, Lublin FD, Miller DH, O'Connor PW, Phillips JT, Polman CH, Radue EW, Rudick RA, Stuart WH, Wajgt A, Weinstock-Guttman B, Wynn DR, Lynn F, Panzara MA (2009). The efficacy of natalizumab in patients with relapsing multiple sclerosis: subgroup analyses of AFFIRM and SENTINEL. J Neurol.

[11] Kapoor R, Ho PR, Campbell N, Chang I, Deykin A, Forrestal F, Lucas N, Yu B, Arnold DL, Freedman MS, Goldman MD, Hartung HP, Havrdova EK, Jeffery D, Miller A, Sellebjerg F, Cadavid D, Mikol D, Steiner D (2018). Effect of natalizumab on disease progression in secondary progressive multiple sclerosis (ASCEND): a phase 3, randomised, double-blind, placebo-controlled trial with an open-label extension. Lancet Neurol.

[12] Devonshire V, Havrdova E, Radue EW, O'Connor P, Zhang-Auberson L, Agoropoulou C, Haring DA, Francis G, Kappos L (2012). Relapse and disability outcomes in patients with multiple sclerosis treated with fingolimod: subgroup analyses of the double-blind, randomised, placebo-controlled FREEDOMS study. Lancet Neurol.

[13] Miller AE, O'Connor P, Wolinsky JS, Confavreux C, Kappos L, Olsson TP, Truffinet P, Wang L, D'Castro L, Comi G, Freedman MS (2012). Pre-specified subgroup analyses of a placebo-controlled phase III trial (TEMSO) of oral teriflunomide in relapsing multiple sclerosis. Mult Scler.

[14] Poser CM, Paty DW, Scheinberg L, McDonald WI, Davis FA, Ebers GC, Johnson KP, Sibley WA, Silberberg DH, Tourtellotte WW (1983). New diagnostic criteria for multiple sclerosis: guidelines for research protocols. Ann Neurol.

[15] Li DK, Paty DW, UBC MS/MRI Analysis Research Group, PRISMS Study Group (1999). Magnetic resonance imaging results of the PRISMS trial: a randomized, double-blind, placebo-controlled study of interferon-beta1a in relapsing-remitting multiple sclerosis. Ann Neurol.

[16] PRISMS Study Group (2001). PRISMS-4: long-term efficacy of interferon-beta-1a in relapsing MS. Neurology.

[17] Li DK, Zhao GJ, Paty DW, The UBC MS/MRi Study Group, The SPECTRIMS Study Group (2001). Randomized controlled trial of interferon-beta-1a in secondary progressive MS: MRI results. Neurology.

[18] Lublin FD, Baier M, Cutter G (2003). Effect of relapses on development of residual deficit in multiple sclerosis. Neurology.

[19] Hirst C, Ingram G, Pearson O, Pickersgill T, Scolding N, Robertson N (2008). Contribution of relapses to disability in multiple sclerosis. J Neurol.

[20] Sormani MP, Li DK, Bruzzi P, Stubinski B, Cornelisse P, Rocak S, De SN (2011). Combined MRI lesions and relapses as a surrogate for disability in multiple sclerosis. Neurology.

[21] Tremlett H, Yousefi M, Devonshire V, Rieckmann P, Zhao Y (2009). Impact of multiple sclerosis relapses on progression diminishes with time. Neurology.

[22] Confavreux C, Vukusic S, Moreau T, Adeleine P (2000). Relapses and progression of disability in multiple sclerosis. N Engl J Med.

[23] Davies F, Edwards A, Brain K, Edwards M, Jones R, Wallbank R, Robertson NP, Wood F (2015). 'You are just left to get on with it': qualitative study of patient and carer experiences of the transition to secondary progressive multiple sclerosis. BMJ Open.

[24] Kappos L (2004). Effect of drugs in secondary disease progression in patients with multiple sclerosis. Mult Scler.

[25] European Study Group on Interferon Beta-1b in Secondary Progressive MS (1998). Placebo-controlled multicentre randomised trial of interferon beta-1b in treatment of secondary progressive multiple sclerosis. Lancet.

[26] Panitch H, Miller A, Paty D, Weinshenker B (2004). Interferon beta-1b in secondary progressive MS: results from a 3-year controlled study. Neurology.

[27] Novartis Pharmaceuticals Corporation (2019) Mayzent (siponimod) US prescribing information. https://www.pharma.us.novartis.com/sites/www.pharma.us.novartis.com/files/mayzent.pdf. Accessed 4 Sept 2019

[28] EMD Serono, Inc. (2019) Mavenclad (cladribine) US prescribing information. https://www.emdserono.com/content/dam/web/corporate/non-images/country-specifics/us/pi/mavenclad-pi.pdf. Accessed 4 Sept 2019

[29] Giovannoni G, Comi G, Cook S, Rammohan K, Rieckmann P, Soelberg SP, Vermersch P, Chang P, Hamlett A, Musch B, Greenberg SJ (2010). A placebo-controlled trial of oral cladribine for relapsing multiple sclerosis. N Engl J Med.

[30] Rammohan K, Giovannoni G, Comi G, Cook S, Rieckmann P, Soelberg Sorensen P, Vermersch P, Hamlett A, Kurukulasuriya N, Clarity Study Group (2012). Cladribine tablets for relapsing-remitting multiple sclerosis: efficacy across patient subgroups from the phase III CLARITY study. Mult Scler Relat Disord.

[31] Giovannoni G, Cook S, Rammohan K, Rieckmann P, Sorensen PS, Vermersch P, Hamlett A, Viglietta V, Greenberg S (2011). Sustained disease-activity-free status in patients with relapsing-remitting multiple sclerosis treated with cladribine tablets in the CLARITY study: a post-hoc and subgroup analysis. Lancet Neurol.

[32] Biogen Inc (2019) Tecfidera (dimethyl fumarate) US prescribing information. https://www.tecfidera.com/content/dam/commercial/tecfidera/pat/en_us/pdf/full-prescribing-info.pdf. Accessed 4 Sept 2019

[33] Biogen Inc (2019) Plegridy (peginterferon beta-1a) US prescribing information. https://www.plegridy.com/content/dam/commercial/multiple-sclerosis/plegridy/pat/en_us/pdf/plegridy-prescribing-information.pdf. Accessed 4 Sept 2019

[34] Weideman AM, Tapia-Maltos MA, Johnson K, Greenwood M, Bielekova B (2017). Meta-analysis of the age-dependent efficacy of multiple sclerosis treatments. Front Neurol.

