# Supplementary figures and images for: Clinical and MRI efficacy of sc IFN β-1a tiw in patients with relapsing MS appearing to transition to secondary progressive MS: post hoc analyses of PRISMS and SPECTRIMS
Source: J Neurol. 2019 Sep 26;267(1):64–75. doi: 10.1007/s00415-019-09532-5 (PMC6954891; doi:10.1007/s00415-019-09532-5)

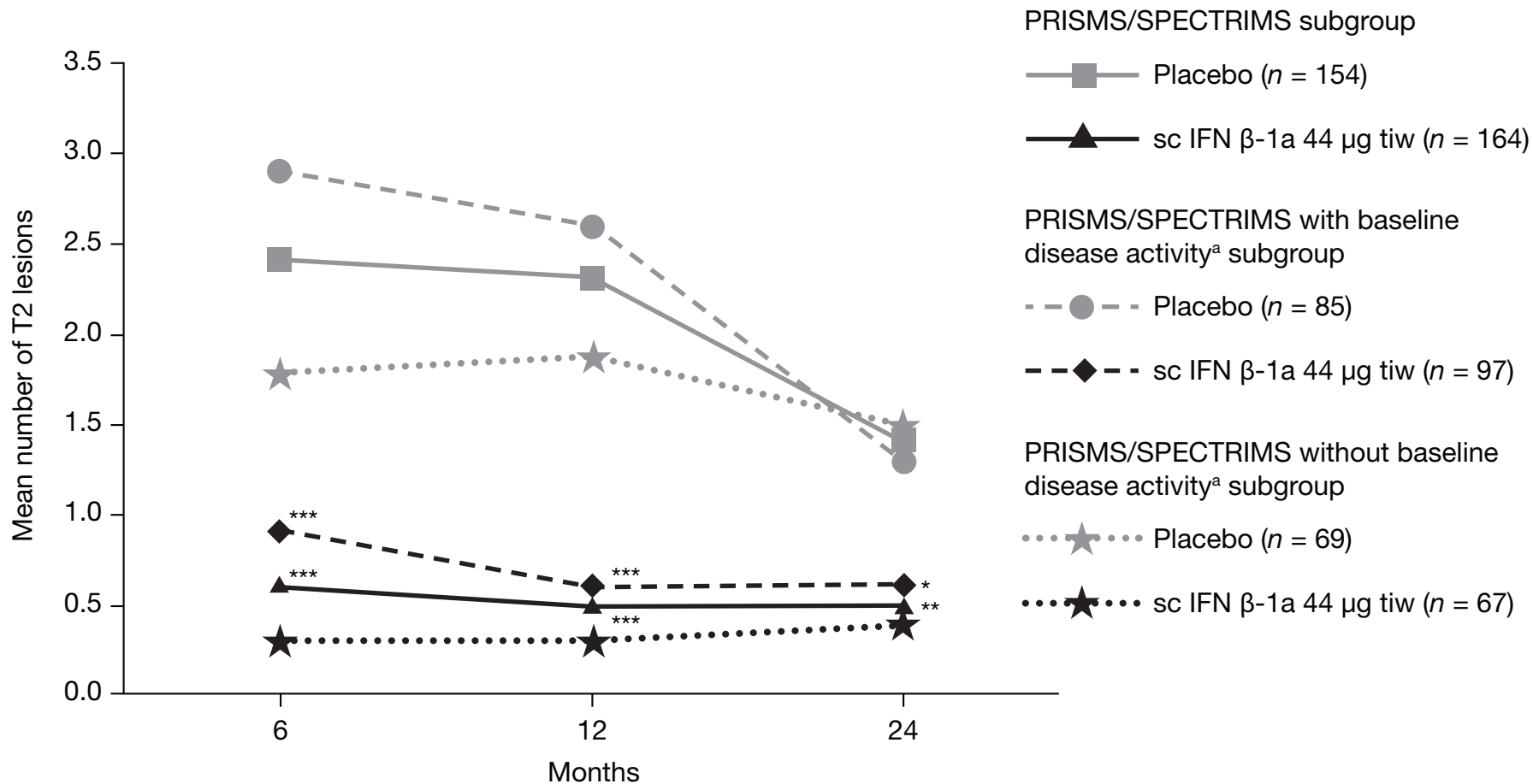

Supplement: Supplementary file 1 — Supplementary file1 (PDF 801 kb) [file 415_2019_9532_MOESM1_ESM.pdf]
